# Does Interpersonal Interaction Really Improve Emotion, Sleep Quality, and Self-Efficacy among Junior College Students?

**DOI:** 10.3390/ijerph17124542

**Published:** 2020-06-24

**Authors:** Po-Yu Wang, Pin-Hsuan Lin, Chung-Ying Lin, Shang-Yu Yang, Kai-Li Chen

**Affiliations:** 1 Department of Pediatric Emergency, Changhua Christian Children Hospital, Changhua 500, Taiwan; dama0115@yahoo.com.tw; 2 Department of Health and Beauty, Shu Zen Junior College of Medicine and Management, Kaohsiung 821, Taiwan; pinhsuan12@ms.szmc.edu.tw; 3 Department of Rehabilitation Sciences, Faculty of Health and Social Sciences, The Hong Kong Polytechnic University, Hung Hom, Hong Kong; cylin36933@gmail.com; 4 Department of Healthcare Administration, College of Medical and Health Science, Asia University, Taichung 413, Taiwan; 5 Department of Nursing, College of Pharmacy and Health Care, Tajen University, Pingtung 90741, Taiwan; tajen2005@yahoo.com.tw

**Keywords:** depression, sleep quality, self-efficacy, interpersonal interaction

## Abstract

This study discusses the correlation between teenagers’ real-life interpersonal interactions and teenagers’ online interpersonal interactions with regards to emotion, sleep quality, and self-efficacy. This study adopted a cross-sectional design that included a survey using a structured questionnaire which included demographic data, the Chinese version of the Depression Anxiety Stress Scale (DASS-21), the Pittsburgh Sleep Quality Index (PSQI), the General Self-Efficacy Scale (GSE), the Real Interpersonal Interaction Scale (RIIS), and the Internet Interpersonal Interaction Scale (IIIS). This study enlisted 917 teenage students (age = 17.16 ± 1.48 years). The study found that RIIS had significant negative correlations with DASS and PSQI scores and a significant positive correlation with GSE. Namely, the greater the degree of real-life interpersonal interaction, the lower the degree of negative emotion. Likewise, the more satisfactory sleep quality is, the higher self-efficacy is. In addition, IIIS scores demonstrate significantly positive correlations with DASS and PSQI scores. Therefore, the greater the degree of online interpersonal interaction, the greater the levels of negative emotion, and the poorer the sleep quality is. This study showed that online interpersonal interaction may not improve emotions, sleep quality, or self-efficacy among junior college students. However, real-life interpersonal interaction may improve those three parameters.

## 1. Introduction

Based on Bowlby’s attachment theory [1], the interaction between an individual and his/her parents has an impact on the individual’s interpersonal behavior, mental health, and personality development. Armsden and Greenberg [2] advocated that, for adolescents, besides their parents, peers are also essential for the development of personal relationships. In other words, interaction with peers is also important for a teenager’s development. Interpersonal interactions affect a teenager’s emotions, with more positive interpersonal interactions with peers leading to lower levels of depression and detrimental emotions [3]. By contrast, negative interpersonal interaction with peers or teachers generates higher levels of depression and more harmful emotions [3]. Studies have proven that a teenager’s favorable interpersonal interactions (with peers or parents) have predictive effects on depressed emotions [4,5,6]. Interpersonal interactions are described as face-to-face interactions between people. However, the speed and convenience of the Internet has prompted increasing numbers of teenagers to make friends or to seek support and recognition on the Internet through what is termed social media. Thus, interpersonal interactions on the Internet are on the increase [7]. Overuse of or addiction to the Internet may negatively influence teenagers’ emotions [8]. Studies [8,9,10] have indicated that teenagers’ Internet addiction is significantly correlated with depressed emotions. However, few studies [8,9,10] discuss further the correlations between interpersonal interactions on the Internet and depressed emotions and whether the interpersonal interactions online actually influence teenagers’ emotions.

Teenagers’ poor interpersonal interactions result in substandard sleep quality, shorter sleep time, and even insomnia [11,12,13]. People with poor sleep quality become more erratic and aggressive in interpersonal interactions, thereby forming a vicious cycle [11,12,13]. Poor sleep quality not only decreases neurobehavioral functioning and alertness but also worsens positive crucial behaviors as well as psychological functions required in social interactions [13,14]. This produces problems in interpersonal interactions, such as aggressive behavior [13,14], school and cyber bullying, and intimate partner violence [15]. In Taiwan, approximately 20% of adolescents have sleep quality problems [16]. Although previous studies have already indicated a significant positive correlation between interpersonal interactions and sleep quality [11,12,13], the associations of interpersonal interactions in real life (i.e., face-to-face interactions) and those on the Internet (e.g., social media) with sleep quality remain unclear, particularly in adolescents. What is clear is that interpersonal interaction pressure and sleep problems continue to intensify in teenagers [17]. Thus, more studies are required to clarify these correlations and provide effective strategies for sleep quality improvement.

The concept of self-efficacy is described as an individual’s ability to self-evaluate which activities to attempt (or avoid) and which to continue (or give up). Self-efficacy thus plays a major role in social behavior [18]. Self-efficacy also affects students’ academic achievement. Students with positive self-efficacy experience a more positive learning experience and outcome (e.g., having a positive working relationship with teachers) than those with negative self-efficacy do [19]. Self-efficacy is influenced by external social scenarios and interpersonal interactions [20]. Students can develop self-efficacy through peer learning, and this successful learning experience equips students with more positive self-efficacy [19]. Therefore, on-campus interpersonal relationships substantially influence teenage students. Favorable interpersonal interactions provide recognition from peers and teachers, and self-efficacy for independent learning promotes positive development [21]. Moreover, a previous study reported that adolescents’ self-efficacy levels can be predicted by variables such as the level of the adolescents’ attachment to their peers and to their parents [22]. Unfortunately, studies on interpersonal interactions and self-efficacy among teenagers remain extremely limited.

In Taiwan, 37.2% of the adolescent population has experienced episodes of depressive mood [8]. Teachers and counselors increasingly encounter teenage students with depression, emotional problems that prompt suicidal thoughts, low self-esteem, and poor sleep quality [23,24,25]. Poor sleep quality in teenagers, causes behavioral problems, such as substance abuse and Internet addiction [16,26,27]. Nevertheless, internet addiction is only under consideration for inclusion in the next Diagnostic and Statistical Manual of Mental Disorder [28]. Furthermore, students with poor self-efficacy often encounter more obstacles with learning [29]. Therefore, determining the factors influencing teenagers’ negative emotion (i.e., depression), sleep quality, and poor self-efficacy has become critical. In summary, the main purpose of this study is to examine the associations of teenagers’ interpersonal interactions, both in real life and on the Internet, with depression, sleep quality, and self-efficacy.

Based on the results of our literature review, we propose three hypotheses: Compared with adolescents with relatively poor (frequency of) interpersonal interactions, both in real life and on the Internet, those with more congenial interpersonal interactions would exhibit better emotion (Hypothesis 1), sleep quality (Hypothesis 2), and self-efficacy (Hypothesis 3).

Although the consistency of one’s behavior is known to be influenced by personal and environmental factors, there is no evidence showing significant behavioral differences between interpersonal-interactions in real life and those on the Internet (social media) [30]. Moreover, studies of interpersonal interactions on the Internet among adolescents, is very limited. Therefore, we assumed no significant difference between interpersonal interactions in real life and those on the Internet in terms of their impact on emotion, sleep quality and self-efficacy.

## 2. Materials and Methods

### 2.1. Study Design and Subject Recruitment

This study adopted a cross-sectional design and used a structured questionnaire for surveys. The collection site was a junior college in southern Taiwan, during the period from 1 May 2019 to 31 July 2019. The students were recruited on a voluntary basis after being given full explanation regarding the purpose of the study by the research assistants of the project. The students were required to complete a paper questionnaire after signing an informed consent form. Eligible participants were taken to a quiet classroom to fill out the questionnaire. In addition, students under 18 years of age provided their consent together with consent from their guardians. The inclusion criteria were as follows: The ability to communicate in Mandarin and complete the questionnaire. The exclusion criteria, as explained by the research assistants to the participants, were as follows: History and/or diagnosis of mental illness or its symptoms, being a parent, and/or working at night. The latter two exclusion criteria were based on the findings of previous studies that being a parent, and/or working at night may affect an individual’s sleep quality and/or interpersonal interactions [31,32,33]. Ethical approval for the study was obtained from the National Cheng Kung University Human Research Ethics Committee (NCKU HREC-E-108-032-2).

### 2.2. Questionnaire

The study’s questionnaire consisted of six sections. The first part was basic demographic information, including sex, age, body mass index, number of days one exercised per week (over 30 min), monthly allowance, relationship status and current residence (home, school dormitory, or off-campus dormitory). The second section is the Chinese version of the Depression Anxiety Stress Scale (DASS-21) developed by Taouk et al., 2001. This scale, which is used for an individual’s self-evaluation of their personal negative emotional behaviors within a one-week period, includes the three aspects (i.e., subscales) of depression, anxiety and stress. Each aspect has 7 questions, with a total of 21 questions. The Likert 4-point scale was adopted, using a scale ranging from 0 points (did not apply to me at all) to 3 points (applied to me very much, or most of the time). The total score was obtained through summation of the scores on the three subscales. The total score ranges from 0 points to 63 points, with higher scores representing higher levels of depression, anxiety, and stress. The Chinese version of the DASS-21 has been validated (Cronbach’s alpha 0.87–0.94) [34]. The Cronbach’s alpha of the total DASS-21 score in this study was 0.95.

The third section of this study’s questionnaire is the Chinese version of Pittsburgh Sleep Quality Index (PSQI). The PSQI was initially developed by Buysee et al. and is used for individuals’ self-evaluation of their sleep quality during a one-month period. The scale consists of seven aspects, including (1) subjective sleep quality, (2) sleep latency, (3) sleep duration, (4) habitual sleep efficiency, (5) sleep disturbances, (6) daytime dysfunction, and (7) use of sleep medication, with a total of 18 questions [35]. A Likert 4-point scale was adopted, and the scores ranged between 0 and 21 points. A higher score indicates poorer sleep quality. The Chinese version of the PSQI has been validated (Cronbach’s alpha 0.87–0.94) [36]. The Cronbach’s alpha of the total PSQI score in this study was 0.81.

The fourth section of the questionnaire is the Chinese version of the General Self-Efficacy Scale (GSE). The GSE was initially developed by Matthias et al. in 1979 and is used for self-evaluation by individuals, and has a total of 10 questions [37]. The Likert 4-point scale was adopted, with its score range from 1 point (Not at all true) to 4 points (Exactly true). The total score ranges from 10 points to 40 points, and a higher score represents more satisfactory self-efficacy. The Chinese version of GSE has been validated (Cronbach’s alpha 0.92) [38]. The Cronbach’s alpha of the total GSE score in this study was 0.92.

The fifth section of the questionnaire is the Real Interpersonal Interaction Scale (RIIS). The RIIS was developed by Chen in 2002. The scale measures the self-evaluated interaction levels of interpersonal relationships in real life (including acquaintances), with a total of 14 questions [39]. RIIS consists of three dimensions, including “intimacy with parents” (6 items), “intimacy with friends” (4 items), and “informative disclosure with friends” (4 items). The Likert 4-point scale was adopted, and the scores range from 1 point “never” to 4 points “often” to explore the frequency of interpersonal interactions in real life. The total score ranges from 14 points to 56 points, and a higher total score represents more intimate interpersonal relationships in real life. Psychometric analysis has shown satisfactory reliability and validity of the RIIS (Cronbach’s alpha 0.88) [39]. The values of Cronbach’s alpha of the total RIIS score and the three subscales (i.e., intimacy with friends, informative disclosure with friends, and intimacy with parents) in this study were 0.91, 0.81, 0.88, and 0.91, respectively.

The sixth part of the questionnaire is the Internet Interpersonal Interaction Scale (IIIS), which, similar to RIIS, was developed by Chen in 2002. The scale measures the self-evaluated levels of online interpersonal interaction (excluding people met in person and including only people known exclusively from the Internet), with a total of 10 questions. The IIIS consists of two dimensions, including “intimacy with online friends” (6 questions) and “informative disclosure with online friends” (4 questions). The Likert 4-point scale was adopted, with its scores ranging from 1 point (representing “never,”) to 4 points (representing “often”) to assess the frequency of online interpersonal interactions. The total score ranges from 10 points to 40 points, and higher total scores represent more intimate interpersonal relationships with online friends. Psychometric analysis has shown satisfactory reliability and validity of IIIS (Cronbach’s alpha 0.84) [39]. The values of Cronbach’s alpha of the total IIIS score and two subscales (i.e., intimacy with online friends and informative disclosure with online friends) in this study were 0.97, 0.94, and 0.95, respectively.

### 2.3. Statistical Analysis

This study adopted the SPSS 22.0 (Mac OX S version) (IBM Corp., Armonk, NY, USA) for data analysis. First, descriptive statistics were adopted to display basic demographic information. Next, Pearson correlation coefficient analysis was used to explore the correlations among DASS-21, PSQI, GSE, RIIS, and IIIS. Finally, multivariate regression analysis was used to examine the correlations between DASS-21, PSQI, GSE, RIIS, and IIIS. The scores for DASS-21, PSQI, and GSE were input into the multivariate regression model as dependent variables, and RIIS (Model 1) and IIIS (Model 2) scores were input in the model as independent variables; all basic demographic information was inputted as adjusted variables. Because sex [40,41], age [16,42], BMI [43,44], exercise habit [8,45], monthly allowance [46,47], girl/boyfriend [48,49], living place [50,51] might have impacted on psychological distress, sleep quality, and self-efficacy, these variables were controlled during analysis. In addition, before the performance of regression analyses, preliminary analyses were conducted to ensure the absence of violation against the assumptions of normality, linearity and multi-collinearity.

## 3. Results

Based on the results of a previous study [52], more than 140 subjects were needed. Accordingly, 919 students in total were enrolled for the present study. After the exclusion of two participants due to questionnaire incompleteness, a total of 917 valid questionnaires were used for analyses (i.e., 99.8% response rate). The participants’ basic information is shown in Table 1, with an average age of 17.16 (±1.48) years. More than half of the participants (56.2%) performed physical exercise more than two days a week, and more than half (55.5%) received less than NT$4000 for their monthly allowance. More than 70% of the participants were not in a relationship, and over 70% lived at home. The results of the Pearson correlation coefficient analysis indicate that the correlation coefficients of DASS-21, PSQI, and GSE with RIIS are −0.20 (*p* < 0.01), −0.14 (*p* < 0.01), and 0.25 (*p* < 0.01), respectively. Their correlation coefficients with IIIS are 0.19 (*p* < 0.01), 0.10 (*p* < 0.01), and 0.01 (*p* = 0.84), respectively.

The multivariate regression results of DASS-21 with RIIS and IIIS scores are shown in Table 2. For RIIS (RIIS model), among the demographic variables, older age and two to three days of exercise per week (Ref: 0–1 days) had significant negative correlations with DASS-21 scores. School dormitory accommodation (Ref: Home) had a significant positive correlation with DASS-21 scores. Regression analysis showed that RIIS was a significant variable for DASS-21 (B: −0.27, S.E.: 0.05, *p* < 0.01). Namely, higher interaction levels for real-life interpersonal relationships imply less prevalent negative emotions. For IIIS (IIIS model), among the demographic variables, older age and two to three days of exercise per week (Ref: 0–1 days) had a significant negative correlation with DASS-21 scores. Living in a school dormitory (Ref: Home) has a significant positive correlation with DASS-21 scores. Regression analysis demonstrated that IIIS was a significant variable for DASS-21 (B: 0.29, S.E.: 0.05, *p* < 0.01), indicating a positive association between the interaction level of interpersonal relationships and the prevalence of negative emotions.

The regression results of PSQI with RIIS and IIIS are shown in Table 3. For RIIS (RIIS model), of the demographic variables, being in a relationship (Ref: None) and living in a school dormitory (Ref: Home) were significantly positively correlated with PSQI scores. Regression analysis demonstrated that RIIS was a significant variable for PSQI (B: −0.05, S.E.: 0.01, *p* < 0.01). Namely, the higher the teenager’s interaction in real-life interpersonal relationships was, the more satisfactory sleep quality was. For IIIS (IIIS model), of the demographic variables, having a romantic partner (Ref: None) and living in a school dormitory (Ref: Home) exhibited significant positive correlations with PSQI scores. The results of the regression analysis showed that IIIS was a significant variable for PSQI (B: 0.04, S.E.: 0.01, *p* < 0.01). Namely, the higher the interaction levels for online interpersonal relationships were, the poorer sleep quality became.

Table 4 shows the regression results for GSE scores with RIIS and IIIS scores. For RIIS (RIIS model), in the demographic variables, monthly allowance exhibited a significant positive correlation with GSE scores. Regression analysis identified RIIS as a significant variable for GSE (B: 0.17, S.E.: 0.02, *p* < 0.01). Namely, the higher the interaction levels for real-life interpersonal relationships were, the more satisfactory the self-efficacy was. For IIIS (IIIS model), in the demographic variables, monthly allowance exhibits a significant positive correlation with GSE scores. The results of regression analysis showed no significant correlation between IIIS and GSE.

## 4. Discussion

The results of this study indicated that real-life interpersonal interactions were significantly correlated with emotions (i.e., depression), sleep quality, and self-efficacy. On the other hand, interpersonal interactions on the Internet exhibited significant correlations with levels of negative emotion and sleep quality. In other words, interpersonal interactions, whether in real life or on the Internet, could significantly influence a teenager’s physical and mental well-being. Although this study revealed significant associations of interpersonal interactions (in real life and online) with emotion, sleep quality, and self-efficacy, the cause-and-effect relationships cannot be confirmed. All the above independent variables might in turn affect interpersonal interactions. However, we believe that our results could provide some initial evidence for future studies to investigate the issue of real-life interpersonal interactions versus Internet interpersonal interactions.

Interpersonal interactions are a crucial part of the self-development process for teenagers, and every teenager requires interpersonal interactions [53]. Among adolescents, their dependence on parents is gradually reduced and transferred to peers, from whom they seek recognition. Favorable relationships with peers or family members can help teenagers to develop positive values and attitudes [54]. On Internet platforms, teenagers can choose how to present themselves; their personal profiles, pictures, and relationships can be edited. The images displayed by individuals can be modified and/or controlled. This type of interaction provides teenagers—who seek intimate relationships but also fear being hurt emotionally—opportunities to accelerate interpersonal interactions through intimate online interactions from a “safe” distance. However, this can also become popular with people of differing opinions which can stifle dialogue [55].

In addition, this study found that higher real-life interpersonal interaction levels reduced depression, anxiety, and stress (Table 2). Higher online interpersonal interaction levels aggravated depression, anxiety, and stress. Teenagers with higher levels of real-life interpersonal interaction mostly had more favorable social skills, emotional regulation, and were psychologically more healthy [56,57]. Thus, they are more likely to receive positive feedback from peers in real life and continue to display positive emotions and behaviors, creating a cycle of positive interpersonal interactions [56,57]. However, teenagers with depression may have poorer interpersonal relationships, social skills, and low self-esteem. Thus, the social setting easily transfers from real life to the Internet because the Internet enables concealment, due to the low appearance factor, and social predominance. A protected platform is provided that allows these teenagers to immerse themselves in the online world [8,58]. However, teenagers with depression easily perceive messages from others as negative messages, even while interacting on the Internet. For example, some messages can be interpreted as “objections” or “opposition” and trigger negative emotions, causing a vicious cycle [59].

This study found a positive association between the level of real-life interpersonal interaction and sleep quality, and found a negative association between the level of online interpersonal interaction and sleep quality. Previous studies have demonstrated that interpersonal interactions among teenagers are significantly correlated with sleep quality [16,27,60,61]. Earlier studies found that interpersonal relationship tension or interpersonal demands result in shorter sleep times and lower sleep quality for teenagers [11]. Furthermore, the interpersonal interaction functions of teenagers are significantly related to emotional regulation abilities [57] (similar results to those in Table 2). Emotional regulation abilities also influence sleep quality [62]. This means that teenagers with higher levels of real-life interpersonal interaction may experience less interpersonal pressure and more favorable emotional regulation and, thus, more favorable sleep quality. Notably, the intimacy of interpersonal relationships with online friends was negatively associated with sleep quality (Table 4). In addition to the findings of other studies, this study further disclosed the correlation between interpersonal interactions on the Internet and sleeps quality, and observed the opposite results from those of “real-life interpersonal interactions.” One of the major reasons speculated for the cause of this is the timing of interactions. The time teenagers interact with online friends is mostly in the evening, particularly before sleep, and sleep problems such as reduced sleeping time and difficulty falling asleep are the likely consequences [16,27]. However, the size of the present study was small thus other factors may influence sleep quality of participants. Therefore, caution is needed when interpreting the results.

This study also indicates that teenagers with higher levels of interpersonal interaction in real life had more favorable self-efficacy. Interpersonal interaction requires regulation of stress and emotions that interpersonal relationships entail [11,57]. Therefore, in the process of interacting with peers, if teenagers can learn qualities required for positive interpersonal interaction such as respect, sincerity, proactivity, praise, self-knowledge, self-acceptance, and life-goal setting, their self-efficacy would benefit [63]. Improved self-efficacy helps to regulate conflicts and stress in interpersonal interactions and causes a virtuous cycle [64]. However, the present study showed that the level of interpersonal interactions on the Internet was not significantly correlated with self-efficacy (Table 4). This may imply that the elements learned in real-life interpersonal interactions, such as self-understanding, life-goal setting, or stress regulation, are more difficult to learn through interpersonal interactions on the Internet.

This study has a few limitations. First, all of the participants were from the same school, and the study was cross-sectional. This rendered study speculations more conservative, and the cause-and-effect relationships cannot be confirmed. Because of its cross-sectional design, all the proposed outcomes might in turn affect interpersonal interactions. In addition, we recruited the participants from multiple classrooms, unfortunately, this study did not collect the participants’ classes and correct possible bias. Thus, the aggregate effect may be performed. Second, all measurements that this study performed on emotions, sleep quality, self-efficacy, and interpersonal interactions were from self-administered scales. Although these scales have been validated and widely used in other studies, they nonetheless cannot accurately/appropriately represent real [human] behavior and some bias (e.g., recall bias, social desirability bias) may have occurred. In addition, both RIIS and IIIS did not investigate the quality of interpersonal interactions. It is recommended that future researchers should take the quality of interpersonal interactions into account. Third, all the multiple regression models showed a low R^2^ (0.03–0.09), indicating limited explanatory power (low model explanation). Fourth, generally speaking, self-efficacy refers to a person’s belief regarding their ability to succeed in specific situations; it is more like an internal mental state and self-perception or self-regulation [65,66]. Whether a trait construct like GSE is theoretically comparable to sleep problems and psychological distress remains to be elucidated. Although previous studies [67,68,69] reported the relationship between GSE and behavioral performance (or changes) as well as the finding that GSE could be an outcome variable in the field of adolescent behavioral research, GSE and human behavior are often interactive and may be inseparable from each other. Therefore, we need to be careful about the interpretation of the results and avoid unnecessary causal inference. Fifth, this study was an exploratory study and focused on exploring the association between interpersonal interaction (both in real life and on the Internet), with emotion, sleep quality, and self-efficacy among junior college students, but gender difference may be in existence. Thus, future studies are recommended to explore the gender differences. Finally, because no standard instrument or measure was used to assess the emotional status of the participants before their enrollment, the possibility still existed that some may have potential undiagnosed psychiatric conditions. Despite the aforementioned limitations, much important information related to teenagers’ health behavior was disclosed.

## 5. Conclusions

This study showed the association between teenagers’ interpersonal interactions in real life and teenagers’ interpersonal interactions on the Internet with regard to their emotional state, their sleep quality, and their self-efficacy. The results showed that higher real-life interpersonal interaction levels were related to reduced negative emotion, improved sleep quality, and more favorable self-efficacy, while higher interpersonal interaction levels on the Internet were associated with higher negative emotional levels and poorer sleep quality. Therefore, negative emotion reduction, sleep quality improvements, and self-efficacy may feasibly be achieved through the enhancement of teenagers’ interpersonal interaction skills and real-life interpersonal interaction opportunities. In addition, this study also revealed that age, number of days one exercised, and location of residence were significant variables for depression. Romantic relationship status and residence location were significant variables for sleep quality. Monthly allowance was a significant variable for self-efficacy.

## Figures and Tables

**Table 1:** Participants’ basic information.

	Total
	N = 917
Sex	
Male	366 (39.9%)
Female	551 (60.1%)
Age (mean ± SD)	17.16 ± 1.48
BMI (mean ± SD)	20.76 ± 3.98
Exercise per week	
0–1 days	401 (43.7%)
2–3 days	325 (35.4%)
≥4 days	191 (20.8%)
Money can be spent each month	
<4000 NTD	509 (55.5%)
4000–5999 NTD	207 (22.6%)
6000–7999 NTD	88 (9.6%)
≥8000 NTD	113 (12.3%)
Have a boy/girl friend (Ref: None)	
No	647 (70.6%)
Yes	270 (29.4%)
Living place	
Home	667 (72.7%)
School dormitory (Ref: home)	110 (12.0%)
Off-campus rental house (Ref: home)	140 (15.3%)
RIIS (mean ± SD)	38.69 ± 8.08
IIIS (mean ± SD)	17.42 ± 7.53
DASS-21 (mean ± SD)	10.82 ± 11.50
PSQI (mean ± SD)	6.27 ± 3.11
GSE (mean ± SD)	23.23 ± 5.50

BMI: Body mass index; NTD: New Taiwan Dollars; SD: Standard Deviation; DASS-21: Depression Anxiety Stress Scales; PSQI: Pittsburgh Sleep Quality Index; GSE: General Self-Efficacy Scale; RIIS: Real Interpersonal Interaction Scale; IIIS: Internet Interpersonal Interaction Scale.

**Table 2:** Multiple regression analysis on factors affecting psychological distress (i.e., Depression, Anxiety, Stress Scale; DASS-21) by interpersonal interaction (real and internet).

	RIIS Model					IIIS Model				
	B	SE	Beta	95% CI	*p*	B	SE	Beta	95% CI	*p*
Independent variable										
Sex (Ref: Male)	−1.05	0.76	−0.05	−2.54,0.45	0.17	−1.12	0.76	−0.05	−2.62,0.37	0.14
Age	−0.74	0.28	−0.10	−1.28,−0.19	<0.01 *	−0.74	0.28	−0.10	−1.29,−0.20	<0.01 *
BMI	−0.06	0.09	−0.02	−0.24,0.14	0.53	−0.13	0.10	−0.05	−0.32,0.06	0.17
Exercise habit (Ref: 0–1 days)										
2–3 days	−1.91	0.84	−0.08	−3.56,−0.25	0.02 *	−2.08	0.84	−0.09	−3.73,−0.43	0.01 *
≥4 days	−1.09	0.99	−0.04	−3.03,0.86	0.27	−1.40	0.99	−0.05	−3.34,0.54	0.16
Monthly allowance	0.18	0.39	0.02	−0.58,0.95	0.64	0.18	0.39	0.02	−0.59,0.94	0.65
Girl/boyfriend (Ref: None)	0.95	0.85	0.04	−0.72,2.61	0.26	0.84	0.85	0.03	−0.83,2.51	0.32
Living place (Ref: Home)										
School dormitory	2.68	1.19	0.08	0.34,5.01	0.02 *	2.37	1.19	0.07	0.03,4.71	0.05 *
Off-campus housing	1.15	1.12	0.04	−1.05,3.35	0.31	1.23	1.12	0.04	−0.97,3.44	0.27
RIIS	−0.27	0.05	−0.19	−0.36,−0.18	<0.01 *	--	--	--	--	--
IIIS	--	--	--	--	--	0.29	0.05	0.19	0.19,0.38	<0.01 *
F-value	6.43 *					6.23 *				
R^2^	0.07					0.06				
Adjusted R^2^	0.06					0.05				

* *p* < 0.05; B: regression coefficient; S.E.: standard error; CI: confidence interval; DASS-21: Depression Anxiety Stress Scales; RIIS: Real Interpersonal Interaction Scale; IIIS: Internet Interpersonal Interaction Scale.

**Table 3:** Multiple regression analysis on factors affecting sleep quality (i.e., Pittsburgh Sleep Quality Index; PSQI) by interpersonal interaction (real and internet).

	RIIS Model					IIIS Model				
	B	SE	Beta	95% CI	*p*	B	SE	Beta	95% CI	*p*
Independent variable										
Sex (Ref: Male)	−0.31	0.21	−0.05	−0.72,0.10	0.14	−0.34	0.21	−0.05	−0.75,0.07	0.11
Age	0.00	0.08	0.00	−0.15,0.15	0.98	−0.00	0.08	0.00	−0.15,0.15	1.00
BMI	0.10	0.03	0.01	−0.05,0.06	0.69	−0.00	0.03	0.00	−0.05,0.05	0.96
Exercise habit (Ref: 0–1 days)										
2–3 days	−0.04	0.23	−0.01	−0.49,0.42	0.88	−0.08	0.23	−0.01	−0.54,0.37	0.72
≥4 days	−0.30	0.27	−0.04	−0.83,0.24	0.28	−0.35	0.27	−0.05	−0.89,0.18	0.19
Monthly allowance	0.14	0.11	0.05	−0.07,0.35	0.19	0.14	0.11	0.05	−0.07,0.35	0.19
Girl/boyfriend (Ref: None)	0.63	0.23	0.09	0.17,1.09	<0.01 *	0.61	0.23	0.09	0.16,1.07	<0.01 *
Living place (Ref: Home)										
School dormitory	0.75	0.33	0.08	0.11,1.39	0.02 *	0.71	0.33	0.07	0.07,1.36	0.03 *
Off-campus rental house	−0.11	0.31	−0.01	−0.72,0.49	0.72	−0.11	0.31	−0.01	−0.72,0.50	0.73
RIIS	−0.05	0.01	−0.13	−0.08,−0.03	<0.01 *	--	--	--	--	--
IIIS	--	--	--	--	--	0.04	0.01	0.09	0.01,0.06	<0.01 *
F-value	4.05 *					3.10 *				
R^2^	0.04					0.03				
Adjusted R^2^	0.03					0.02				

* *p* < 0.05; B: regression coefficient; S.E.: standard error; CI: confidence interval; PSQI: Pittsburgh Sleep Quality Index; RIIS: Real Interpersonal Interaction Scale; IIIS: Internet Interpersonal Interaction Scale.

**Table 4:** Multiple regression analysis on factors affecting self-efficacy (i.e., General Self-Efficacy Scale; GSE) by interpersonal interaction (real and internet).

	RIIS Model					IIIS Model				
	B	SE	Beta	95% CI	*p*	B	SE	Beta	95% CI	*p*
Independent variable										
Sex (Ref: Male)	−0.47	0.36	−0.04	−1.18,0.24	0.19	−0.27	0.37	−0.02	−1.00,0.46	0.47
Age	0.21	0.13	0.06	−0.05,0.47	0.11	0.22	0.14	0.06	−0.05,0.49	0.11
BMI	0.05	0.05	0.04	−0.04,0.14	0.27	0.05	0.05	0.03	−0.04,0.14	0.31
Exercise habit (Ref: 0–1 days)										
2–3 days	0.29	0.40	0.03	−0.49,1.07	0.47	0.55	0.41	0.05	−0.26,1.35	0.18
≥4 days	0.30	0.47	0.02	−0.62,1.22	0.52	0.50	0.48	0.04	−0.45,1.44	0.30
Monthly allowance	0.79	0.18	0.15	0.42,1.15	<0.01 *	0.78	0.19	0.15	0.40,1.15	<0.01 *
Girl/boyfriend (Ref: None)	−0.42	0.40	−0.04	−1.21,0.37	0.30	−0.40	0.41	−0.03	−1.21,0.41	0.33
Living place (Ref: Home)										
School dormitory	0.19	0.56	0.01	−0.91,1.30	0.73	0.17	0.58	0.10	−0.97,1.31	0.77
Off-campus rental house	−0.94	0.53	−0.06	−1.98,0.11	0.08	−0.87	0.55	−0.06	−1.95,0.20	0.11
RIIS	0.17	0.02	0.25	0.13,0.21	<0.01 *	--	--	--	--	--
IIIS	--	--	--	--	--	−0.00	0.02	0.00	−0.05,0.05	1.00
F-value	9.03 *					2.70 *				
R^2^	0.09					0.03				
Adjusted R^2^	0.08					0.02				

* *p* < 0.05; B: regression coefficient; S.E.: standard error; CI: confidence interval; GSE: General Self-Efficacy Scale; RIIS: Real Interpersonal Interaction Scale; IIIS: Internet Interpersonal Interaction Scale.
